# CRISPR memories in single cells

**DOI:** 10.15252/msb.202211011

**Published:** 2022-04-25

**Authors:** Anke Sparmann, Chase L Beisel

**Affiliations:** ^1^ Helmholtz Institute for RNA‐based Infection Research (HIRI) Helmholtz‐Centre for Infection Research (HZI) Würzburg Germany; ^2^ Medical Faculty University of Würzburg Würzburg Germany

**Keywords:** Microbiology, Virology & Host Pathogen Interaction

## Abstract

CRISPR‐Cas systems allow bacteria to memorize prior infections as a means to combat the same invader if it attempts another attack in the future. While the underlying mechanisms of this bacterial immunity have been intensely studied over the past decade, little attention has been paid to CRISPR defense at the single‐cell level. In their recent work, Brouns and colleagues (McKenzie *et al*, 2022) track memory acquisition and defense in individual cells and find a wide range of temporal dynamics that shape how a cell population experiences and combats an active infection.

## Graphical abstract

How CRISPR defense plays out in single bacterial cells remains largely unknown. Brouns and colleagues (McKenzie *et al*, 2022) take an important step toward addressing this challenge by studying spacer acquisition and invader clearance at the single‐cell level.

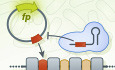

CRISPR‐Cas systems, well‐known as the source of widely used genome editing tools, naturally protect bacteria and archaea from foreign invaders such as bacteriophages or mobile plasmids (Hille *et al*, 2018). Protection starts with each system storing small fragments of DNA derived from the invader within the genome of the host. The stored fragments, called spacers, are then used as templates to transcribe CRISPR RNAs, which direct cleavage of the matching invader DNA to halt the infection. Because spacers are passed to each new generation of cells and can distinguish the invader from other genomic sequences, CRISPR‐Cas systems offer long‐term immunity, not only for the original cell, but also for its future progeny. Despite this ingenious setup, time is of the essence. Cells undergoing an active infection must quickly acquire a spacer and mount a counteroffensive before the invader wipes out the entire cell population. This challenge raises many scientific questions: how quickly can spacers be acquired and then used against the invader? How does this process occur in individual cells? How does this impact a cell population undergoing an active infection? While spacer acquisition and invader defense have been extensively studied on the cellular population level using bulk analytical techniques, little is known how this process plays out in single cells.

Brouns and coworkers take an important step to addressing this challenge by studying spacer acquisition and invader clearance at the single‐cell level (McKenzie *et al*, 2022). They focused on the type I‐E CRISPR‐Cas system in the bacterium *Escherichia coli*, which generally uses two modes of spacer acquisition called naive acquisition and primed acquisition (Fineran *et al*, 2014; Levy *et al*, 2015). Naive acquisition usually incorporates spacers derived from stalled replication forks, but this mode tends to be extremely slow. Primed acquisition is triggered when the CRISPR machinery encounters a spacer target that has accumulated mutations. While these mutations prevent a full‐fledged attack by the CRISPR machinery, the mutated spacer target can drive the acquisition of an adjacent fragment of invader DNA. This mode acts much faster than naive acquisition (Fineran *et al*, 2014).

The authors set out to measure the timing of acquisition and defense at the single‐cell level in a growing population. Using a fluorescent reporter plasmid encoding a perfect or mutated spacer target in a bacterial strain with inducible expression of the CRISPR‐Cas system, the authors were able to follow the loss of the plasmid over time via time‐lapse microscopy (Fig 1). By tagging one component of the CRISPR machinery with another fluorescent protein, the authors could also evaluate how plasmid loss correlated with single‐cell abundance of the machinery. In addition, this approach allowed concomitant assessment of other factors, such as a cell’s doubling time and the fate of progeny with a shared parent (sister cells) or grandparent (cousin cells). Finally, by incorporating stochastic modeling, the authors interrogated mechanistic features difficult to probe through experimental means.

**Figure 1:**
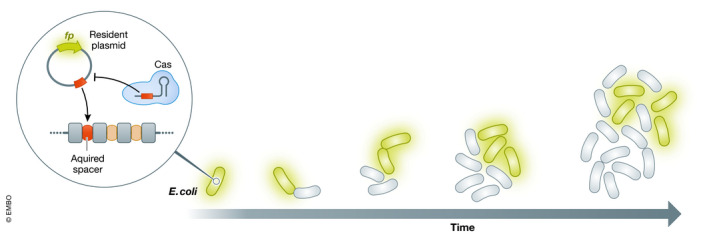
Single‐cell tracking of invader memorization by CRISPR‐Cas systems Time‐lapse microscopy was used to track when single *E. coli* cells gained protection against a resident plasmid and managed to clear it following induction of the CRISPR‐Cas system. The approach revealed that the time needed to acquire protection varied considerably across the cell population and allowed the analysis of contributing factors, with implications for how CRISPR‐Cas systems protect a population of cells against a foreign invader. *fp*: gene encoding a fluorescent protein used to track the presence of the plasmid.

This set of approaches led to intriguing insights that help inform how CRISPR‐based immunity plays out across a population. For one, clearance of the plasmid with the perfect spacer target was rapid (1–3 cell doublings) and relatively uniform across the population, showing that direct interference can offer robust protection to an entire cell population. In contrast, clearance of the plasmid with the mutated spacer target was extremely variable, spanning 2–30 cell doublings. This variability was traced to the process of primed acquisition and the ranging time scales in which a new spacer could be acquired. Because direct interference commences shortly after a new spacer is acquired, the plasmid was much more likely to be cleared at the same time by sister cells than by cousin cells or any other cell in the population. The authors found that the cellular features they tracked had different effects on plasmid clearance and primed acquisition. For example, high levels of the CRISPR machinery correlated with faster clearance but not primed acquisition. Separately, rapid cellular growth was associated with faster clearance but also slower acquisition. In addition, modeling indicated that variability in the levels of the CRISPR machinery could accelerate primed acquisition across the population due to a highly non‐linear relationship between abundance and acquisition. These insights paint a complex picture of the factors influencing CRISPR‐based defense, leading to a hypervariable response if defense occurs through primed acquisition. Such a response would leave most of the cell population susceptible to invader attack, leading to few survivors. The upside is that these survivors carry robust immunity against the invader if a second attack occurs again in the future.

Notwithstanding the authors’ advances, there are some limitations when extrapolating these findings to natural protection by CRISPR‐Cas systems. For instance, the use of an inducible system to express the CRISPR machinery contrasts with natural systems that are constitutively expressed under infection conditions. The use of a plasmid as a target makes it difficult to extrapolate the findings to infection by bacteriophages that rapidly propagate and lyse the cell. Finally, the CRISPR‐Cas system in *E*. *coli* has remained an oddity because it is insufficiently expressed under any natural growth conditions to enact immune defense (Pul *et al*, 2010; Westra *et al*, 2010). While these limitations are justifiable given the experimental setup and the benefits of using a well‐characterized system, the authors’ findings raise important questions that will drive further research into CRISPR‐based defense. What are the single‐cell dynamics during an active infection by lytic bacteriophages often targeted by CRISPR‐Cas systems? How do the dynamics and underlying mechanisms change across the rich diversity of CRISPR‐Cas systems? And how do these dynamics influence the evolution of CRISPR‐Cas systems and invaders, out to circumvent the defense system? By bringing single‐cell techniques into the realm of CRISPR biology, we can expect to learn much more about the functions of these ingenious immune systems and possibly translate these insights into new and improved CRISPR technologies.

## References

[msb202211011-bib-0001] Fineran PC , Gerritzen MJH , Suárez‐Diez M , Künne T , Boekhorst J , van Hijum SAFT , Staals RHJ , Brouns SJJ (2014) Degenerate target sites mediate rapid primed CRISPR adaptation. Proc Natl Acad Sci USA 111: E1629–E1638 24711427 10.1073/pnas.1400071111 PMC4000823

[msb202211011-bib-0002] Hille F , Richter H , Wong SP , Bratovič M , Ressel S , Charpentier E (2018) The biology of CRISPR‐Cas: backward and forward. Cell 172: 1239–1259 29522745 10.1016/j.cell.2017.11.032

[msb202211011-bib-0003] Levy A , Goren MG , Yosef I , Auster O , Manor M , Amitai G , Edgar R , Qimron U , Sorek R (2015) CRISPR adaptation biases explain preference for acquisition of foreign DNA. Nature 520: 505–510 25874675 10.1038/nature14302 PMC4561520

[msb202211011-bib-0004] McKenzie RE , Keizer E , Vink J , van Lopik J , Büke F , Kalkman V , Fleck C , Tans S , Brouns S (2022) Single cell variability of CRISPR‐Cas interference and adaptation. Mol Syst Biol 18: e10680 10.15252/msb.202110680 PMC10561596 35467080

[msb202211011-bib-0005] Pul U , Wurm R , Arslan Z , Geissen R , Hofmann N , Wagner R (2010) Identification and characterization of *E. coli* CRISPR‐cas promoters and their silencing by H‐NS. Mol Microbiol 75: 1495–1512 20132443 10.1111/j.1365-2958.2010.07073.x

[msb202211011-bib-0006] Westra ER , Pul Ü , Heidrich N , Jore MM , Lundgren M , Stratmann T , Wurm R , Raine A , Mescher M , Van Heereveld L *et al* (2010) H‐NS‐mediated repression of CRISPR‐based immunity in *Escherichia coli* K12 can be relieved by the transcription activator LeuO. Mol Microbiol 77: 1380–1393 20659289 10.1111/j.1365-2958.2010.07315.x

